# The application of inductive policies to scientific research publication

**DOI:** 10.5935/0004-2749.2024-1015

**Published:** 2024-12-26

**Authors:** Newton Kara-Junior

**Affiliations:** 1 Ophthalmology Department, Universidade de São Paulo, São Paulo, SP, Brazil; 2 Editor-in-Chief of Arquivos Brasileiros de Oftalmologia

Inductive policies are tools adopted by governments and organizations to influence and direct strategic objectives. These policies are widely used in the healthcare field^(1)^.

With the support of its editors, the Arquivos Brasileiros de Oftalmologia (ABO) aims to develop and implement an inductive policy applicable to scientific research publications. The objective of this is to create scientific research conditions that allow more national researchers to publish their studies in ABO without lowering the quality standards of the journal.

The improved scientific quality of the ABO in recent years has been celebrated by the national ophthalmological community. The existing advantages, and those obtained by the journal, include indexing in the main databases, a good scientific impact factor, open access, no publication fees, and a fast editorial flow. While these benefits brought the bonus of internationalization, they also imposed some restrictions, with publication in the ABO becoming more difficult for national researchers due to foreign competition.

To address this issue and assist national researchers, especially those not affiliated with research institutions, the ABO has initiated a Scientific Mentoring program. In this program, ABO editors evaluate research projects submitted by interested parties and select those with scientific potential and practical viability. Researchers on the selected projects are then provided with individual guidance on conducting their literature review, designing the study methodology, and obtaining ethics committee approval. In the next phase of the program, the ABO guides the execution of the study, the tabulation of the data, the analysis of the results, and the writing of the article.

Articles accepted for publication in the ABO are generally expected to describe new techniques or technologies, to have sufficient sample sizes for statistically viable results, and to have a prospective design. However, innovations and epidemiological studies have also been published. The ABO editors involved in the Scientific Mentoring program know which studies have publication potential and direct their guidance accordingly. As such, this initiative operates as an inductive policy in the sense that ABO editors are inductively selecting the articles that they are interested in publishing.

The Scientific Mentoring program constitutes an unprecedented evolution of preprinting. Preprint databases list articles that have not yet been published in scientific journals. These are available for other researchers to comment on and allow scientific editors interested in the study topic to invite authors to submit a specific article to their journal. Scientific Mentoring-ABO goes further by providing preprint support earlier in the process. By offering assistance from inception to finished article, ABO encourages doctors to research. It should be noted that all articles are still subject to peer review to ensure an impartial assessment of the scientific rigor of the study.

All of ABO’s editors are currently involved in the Scientific Mentoring program. The program has now begun its first phase, with more than 700 interested ophthalmologists nationwide. If the initiative is successful, it will be repeated annually and become an ongoing ABO policy.
